# Modeling aerotaxis band formation in *Azospirillum brasilense*

**DOI:** 10.1186/s12866-019-1468-9

**Published:** 2019-05-17

**Authors:** Mustafa Elmas, Vasilios Alexiades, Lindsey O’Neal, Gladys Alexandre

**Affiliations:** 1 Mathematics, University of Tennessee, 1403 Circle Dr, Knoxville, TN, 37996 USA; 2 Biochemistry and Cellular & Molecular Biology, University of Tennessee, 1311 Cumberland Ave, Knoxville, TN, 37996 USA

**Keywords:** Chemotaxis, Aerotaxis, Band formation, Azospirillum brasilense, Mathematical modeling

## Abstract

**Background:**

Bacterial chemotaxis, the ability of motile bacteria to navigate gradients of chemicals, plays key roles in the establishment of various plant-microbe associations, including those that benefit plant growth and crop productivity. The motile soil bacterium *Azospirillum brasilense* colonizes the rhizosphere and promotes the growth of diverse plants across a range of environments. Aerotaxis, or the ability to navigate oxygen gradients, is a widespread behavior in bacteria. It is one of the strongest behavioral responses in *A. brasilense* and it is essential for successful colonization of the root surface. Oxygen is one of the limiting nutrients in the rhizosphere where density and activity of organisms are greatest. The aerotaxis response of *A. brasilense* is also characterized by high precision with motile cells able to detect narrow regions in a gradient where the oxygen concentration is low enough to support their microaerobic lifestyle and metabolism.

**Results:**

Here, we present a mathematical model for aerotaxis band formation that captures most critical features of aerotaxis in *A. brasilense*. Remarkably, this model recapitulates experimental observations of the formation of a stable aerotactic band within 2 minutes of exposure to the air gradient that were not captured in previous modeling efforts. Using experimentally determined parameters, the mathematical model reproduced an aerotactic band at a distance from the meniscus and with a width that matched the experimental observation.

**Conclusions:**

Including experimentally determined parameter values allowed us to validate a mathematical model for aerotactic band formation in spatial gradients that recapitulates the spatiotemporal stability of the band and its position in the gradient as well as its overall width. This validated model also allowed us to capture the range of oxygen concentrations the bacteria prefer during aerotaxis, and to estimate the effect of parameter values (e.g. oxygen consumption rate), both of which are difficult to obtain in experiments.

## Background

Plant-microbe associations play a vital role in plant health and crop productivity. The ability to detect and respond to environmental changes in the vicinity of bacteria is essential for their survival and growth. A variety of mechanisms have evolved by which cells sense their environmental changes and respond appropriately. One of the best characterized bacterial responses to changes in the environment is chemotaxis, the ability of motile cells to navigate chemical gradients [1]. In chemotaxis, motile bacteria efficiently and rapidly respond to changes in the chemical composition of their environment, moving towards regions with increasing concentrations of favorable chemicals (chemoattractants) and away from regions with increasing concentration of unfavorable chemicals (chemorepellents) by biasing their basal motility pattern. Motility and the ability of bacteria to locate niches that support optimum growth in the rhizosphere by chemotaxis is essential for their survival and enhances their competitiveness in this environment [2, 3].

Aerotaxis is chemotaxis in an oxygen gradient. This bacterial behavior was first reported by Engelmann in 1881. He observed the aggregation of an organism around air bubbles [4, 5]. Beijerinck later confirmed Engelmann’s finding and further described the formation of a sharp band of motile cells, corresponding to their accumulation, around a source of oxygen [6]. He also observed that the band of motile organisms descended when air was replaced with oxygen and ascended when air was replaced with hydrogen, implying that the organisms moved towards a specific concentration of oxygen. The preferred concentration of oxygen in a gradient has been determined for a few motile bacterial species (reviewed in [2]) including 200 *μ*M for *Bacillus subtilis* (an obligate aerobe), 50 *μ*M for *Escherichia coli* (a facultative anaerobe), 0.4 *μ*M for *Desulfovibrio vulgaris* (an aerotolerant anaerobe), and 3-5 *μ*M for *Azospirillum brasilense* (a microaerobe).

There are two types of aerotaxis responses known to date. In aerobes such as *B. subtilis* motile bacteria respond directly to the oxygen concentration and accumulate at the highest concentrations of oxygen in the gradient [7]. In other organisms, such as *E. coli* and *A. brasilense*, cells perform aerotaxis not by sensing oxygen itself, but by monitoring the effects that oxygen has on the metabolism of the cells [8, 9]. This behavior is broadly referred to as **energy taxis** [2]. In energy taxis-based aerotaxis, cells do not move toward the greatest concentration of oxygen but toward an intermediate concentration of oxygen that supports maximum energy levels. The signal for this type of behavior originates within the electron transport system, where oxygen-mediated changes of the rate of electron transport, redox status or proton motive force are detected during aerotaxis [2, 10].

**Fig. 1:**
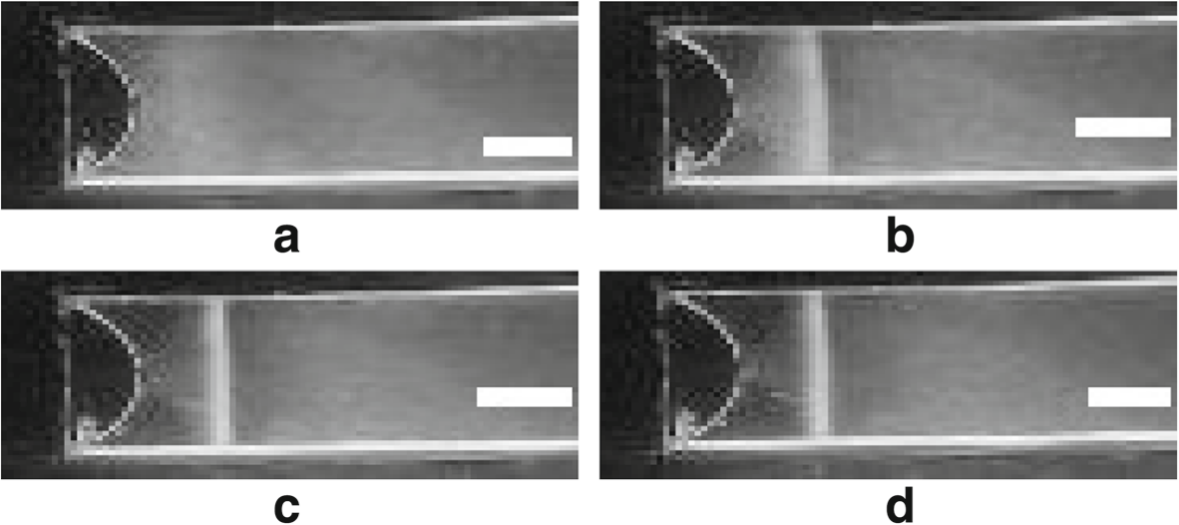
Images of aerotactic band formation of wild-type (Sp7) *A. brasilense* with 21% oxygen set at the meniscus. (**a**) At time 0 sec, when oxygen is applied at the meniscus. (**b**) At time 50 sec. (**c**) At time 100 sec. (**d**) At time 140 sec, by which time the band has already stabilized. Scale bar is 500 *μ*m in all panels

Aerotaxis is a major behavioral response in *A. brasilense* [11], characterized by a remarkable ability to precisely locate niches where oxygen concentrations are low and optimal to support metabolism. At such locations motile cells form sharp bands (as seen in Fig. 1). *A. brasilense* senses very high and low oxygen concentrations as repellents and accumulates at intermediate concentrations, preferring about 5 *μ*M dissolved oxygen [10] (note that 1% of oxygen in air corresponds to 13 *μ*M dissolved oxygen in water). Energy taxis guides *A. brasilense* to move towards microenvironments optimal for maximum energy generation and nitrogen fixation [10, 12]. The location and width of a band are primary observable and measurable quantities in aerotaxis experiments [13].

The motile soil bacterium *Azospirillum brasilense* colonizes the rhizosphere and promotes the growth of a variety of plants across a range of environments. It is 2-3 *μ*m long, with a single polar flagellum, [14]. When the flagellum rotates counterclockwise (CCW), the cell moves forward on a straight line, called a **run**. When the flagellum rotates clockwise (CW), the cell moves backward, and may also change direction, called a **reversal**. The frequency of reversals determines whether the cell moves away (when reversal frequency is low, so runs predominate) or remains nearby (when reversal frequency is high).

A model of aerotactic band formation, incorporating energy taxis, was developed by Mazzag et al. [15]. It is based on earlier models for chemotaxis and aerotaxis, which consider the movement of bacteria in one dimension and distinguishes right- and left-moving cells depending on their swimming direction to the oxygen gradient. While the model [15] captured some of the features of the aerotaxis response of *A. brasilense*, it failed to produce a stable aerotactic band, which is typical of that formed by *A. brasilense*.

**Fig. 2:**
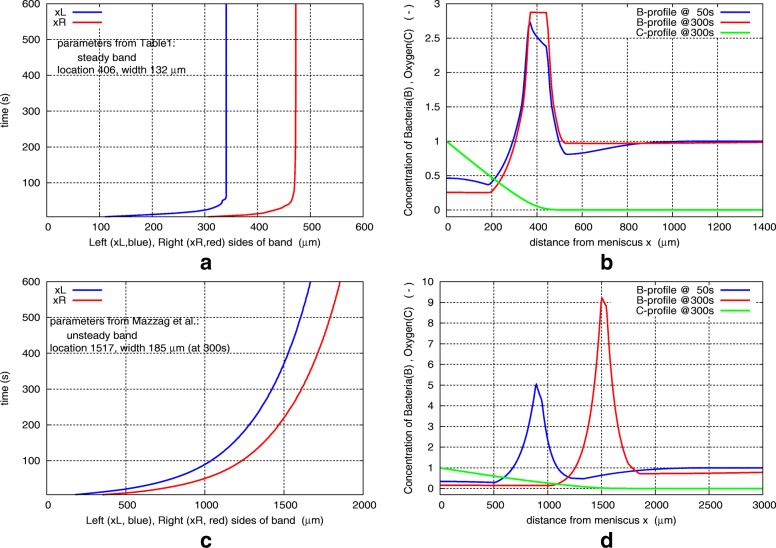
Aerotactic band formation predicted by the model. **Top row**: With parameters of Table 1. The band forms and stabilizes within a minute, and remains steady, exactly as observed in experiments. Band location and width are 406 *μm* and 132 *μm*, in excellent agreement with the experimentally measured values of 407 and 132 *μ*m. **Bottom row**: With parameter values taken from Mazzag et al. [15]. The band is moving (not steady); location and width are 1517 and 185 *μ*m at 300 s, but 1760 and 186 *μ*m at 600 s. (**a**),(**c**): Band evolution in time: Left (blue) and Right (red) sides of the band. Note the different scales on x-axis. (**b**),(**d**): Profiles of (normalized) bacteria concentration (B) at time 50 s (blue) and 300 s (red), and of Oxygen concentration (C) at 300 s (green). Note the different scales on both axes

Here, we use the same basic energy taxis model of Mazzag et al., with some adjustments, enhanced numerical implementation, and use experimentally measured parameter values for *A. brasilense* (Table 1), to recapitulate the aerotactic band formation in this species (Fig. 2). We also present numerical simulations to predict how the *A. brasilense* aerotaxis band would respond to changes in parameters (Table 2). Details on implementation and differences with [15] are given at the end of Mathematical Model section.

**Table 1:** Parameter values for the aerotactic band formation model

Parameter	Description	Value
*B*_*o*_	Total bacteria concentration	7×10^8^ cells/ml
*C*_*o*_	Oxygen concentration at the meniscus	21%
*D*	Oxygen Diffusion coefficient	2×10^3^*μm*^2^/s
*K*	Bacteria oxygen consumption rate	4×10^−9^*μ*M ml/s cell
*v*	Swimming speed	20 *μ*m/s
*F*_*max*, *band*_	maximum reversal frequency inside the band	0.96/s
*F*_*max*_	maximum reversal frequency outside of band	0.65/s
*F*_*min*_	minimum reversal frequency	0.35/s
$\widehat {C}_{max}$	Upper detectable oxygen concentration	10%
*C*_*max*_	Upper favorable oxygen concentration	2%
*C*_*min*_	Lower favorable oxygen concentration	0.3*%*
$\widehat {C}_{min}$	Lower detectable oxygen concentration	0.01*%*
S	Length of the capillary	5 mm

**Table 2:** Sensitivity of band location and width on parameters. Only one parameter is varied at a time, with all others at their base values as in Table 1. For ease of comparison, base value of each parameter is listed, which produces location 406, width 132 *μ*m. The % changes are relative to base values. Columns 4 and 6 show sensitivity of location and width on each parameter. “Bpeak” in column 7 is the maximum bacterial concentration at 300 s (normalized by the initial concentration *B*_*o*_). “Smoothness” in column 8 refers to the appearance of left- and right-side of band location vs time, as seen in Fig. 2

Parameter	% change	Location(*μ*m)	% change	Width(*μ*m)	% change	Bpeak	Smoothness
*B*_*o*_=1×10^9^ cells/ml	+50*%*	352	−13*%*	114	−14%	2.6	Smooth
*B*_*o*_=7×10^8^ cells/ml	base value	406	–	132	–	2.9	Smooth
*B*_*o*_=3×10^8^ cells/ml	−50%	582	+43%	156	+18%	3.9	Smooth
*C*_*o*_=30%	+50%	527	+30%	122	−8%	3.2	∼smooth
*C*_*o*_=21%	base value	406	–	132	–	2.9	Smooth
*C*_*o*_=10%	−50%	227	−44%	133	1%	2.6	smooth >220s
*D*=2.5×10^3^*μm*^2^/s	+25%	449	+11%	140	+6%	3.1	Smooth
*D*=2.0×10^3^*μm*^2^/s	base value	406	–	132	–	2.9	Smooth
*D*=1.5×10^3^*μm*^2^/s	−25%	359	−12%	114	−13%	2.7	Smooth >200s
*K*=6×10^−9^*μ*M ml/s/cell	+50%	344	−15%	114	−14%	2.6	Smooth >100s
*K*=4×10^−9^*μ*M ml/s/cell	base value	406	–	132	–	2.9	Smooth
*K*=2×10^−9^*μ*M ml/s/cell	−50%	547	+32%	149	+13%	3.7	Smooth >110s
*v*= 30 *μ*m/s	+50%	429	+6%	144	+9%	2.4	∼smooth
*v*= 20 *μ*m/s	Base value	406	–	132	–	2.9	Smooth
*v*= 10 *μ*m/s	−50%	382	−6%	98	−26%	4.2	Smooth >200s
*F*_*max*_= 0.85 /s	+30%	387	−5%	125	−5%	3.5	Smooth
*F*_*max*_= 0.65 /s	Base value	406	–	132	–	2.9	Smooth
*F*_*max*_= 0.45 /s	−30%	445	+10%	133	0%	1.9	∼smooth
*F*_*min*_= 0.45 /s	+30%	426	+5%	141	+7%	2.4	Smooth
*F*_*min*_= 0.35 /s	Base value	406	–	132	–	2.9	Smooth
*F*_*min*_= 0.25 /s	−30%	387	−5%	117	−11%	3.5	Smooth
*C*_*max*_= 3%	+50%	383	−6%	159	+21%	2.6	∼smooth
*C*_*max*_= 2%	Base value	406	–	132	–	2.9	Smooth
*C*_*max*_= 1%	−50%	457	+13%	94	−29%	3.4	∼smooth
*C*_*min*_= 0.45%	+50%	391	−4%	115	−13%	3.2	Smooth
*C*_*min*_= 0.3%	Base value	406	–	132	–	2.9	Smooth
*C*_*min*_= 0.15%	−50%	437	+8%	148	+12%	2.5	∼smooth
$\widehat {C}_{max}$= 15%	+50%	402	−1%	126	−5%	3.2	Smooth >120s
$\widehat {C}_{max}$= 10%	Base value	406	–	132	–	2.9	Smooth
$\widehat {C}_{max}$= 5%	−50%	418	+3%	125	−6%	2.4	∼smooth
$\widehat {C}_{min}$= 0.015%	+50%	406	0%	132	0%	2.9	Smooth
$\widehat {C}_{min}$= 0.010%	Base value	406	–	132	–	2.9	Smooth
$\widehat {C}_{min}$= 0.005%	−50%	406	0%	132	0%	2.9	Smooth

The main objective is to validate the model, by showing that it is capable of capturing experimental observations not only qualitatively but also quantitatively.

## Results

This section contains a series of computer simulations of the mathematical model described in section **Mathematical Model**.

The model consists of advection-reaction equations for right-moving and left-moving bacteria in a capillary tube, and a diffusion-reaction equation for oxygen, which diffuses into the water from the meniscus and is consumed by bacteria. The primary computed quantities are *B*(*x*,*t*) and *C*(*x*,*t*), the bacteria and oxygen concentrations at location *x* at time *t*, and the location of the left-side and right-side of the band, found as FWHM (Full Width at Half Maximum) from *B*(*x*,*t*) at each *t*. We plot the evolution of the band in time and profiles of *B* and *C* at desired times, see Fig. 2a b. Some implementation details are given in subsection **Numerical Implementation**.

### Experimental validation on wild type *A. brasilense*

We present here results of simulations of actual experiments with wild type (Sp7) *A. brasilense* grown in malate (as carbon source). In all the simulations, bacteria formed a stable aerotactic band. The location and width of the band agree well with those measured for microaerophilic *A. brasilense* cells in [10, 13].

Experimentally measured band location and width, for cells inoculated into the spatial gradient at the density determined by CFU counts, were respectively 407±168 and 132±44 *μm* at time 300 s. Cell tracking yielded mean values for speed *v*=20 *μ*m/s, reversal frequency before stimulation *F*_*min*_ = 0.35 /s, reversal frequency inside the band *F*_*max*,*band*_ = 0.96 /s, and outside the band *F*_*max*_ = 0.65 /s, at time 300 s.

Using these values in the model, we determined the switch parameters $\widehat {C}_{max}$, *C*_*max*_, *C*_*min*_, $\widehat {C}_{min}$, which determine the forcing terms in the advection eqs. 1, 2 according to 3 and 4 (see **Mathematical Model**), to capture the experimentally measured band location and width. Numerical simulation of the model, with parameter values listed in Table 1, produces band left and right sides at 340.3 and 472.1 *μm*, hence band location (midpoint) 406.2 and width 131.8 *μm*. This is in remarkable agreement with the measured values of 407 and 132 *μm*.

Shown in Fig. 2a is the band evolution up to 600 seconds. Figure 2b shows profiles of bacterial density *B* at times 50 s and 300 s, and of oxygen concentration *C* at 300 s. Note that the bacterial concentration *B*(*x*,*t*) is normalized by the initial value *B*_*o*_, so *B*=1 is the initial concentration (assumed uniform in the capillary). Similarly, *C*(*x*,*t*) is normalized by *C*_*o*_, the oxygen at the meniscus. The rapid formation and stability of the band, seen in Fig. 2a, captures well the experimental observations seen in Fig. 2.

The values of the *C*-switches ($\widehat {C}_{max}$, *C*_*max*_, *C*_*min*_, $\widehat {C}_{min}$) are *effective* concentrations at which switching of reversal frequencies occurs, creating a band. Their values capture the oxygen level the bacteria prefer during aerotaxis, which is difficult to measure experimentally. In the above simulation, the concentration of oxygen along the right-side of the band is *C*=0.09*%*, close to $\widehat {C}_{min}=0.01\%$. Along the middle of the band, *C*=0.88*%* is roughly half-way between *C*_*min*_=0.3*%* and *C*_*max*_=2*%*. And along the left-side, closer to the meniscus, *C*=2.85*%* is just above *C*_*max*_. Thus here the band forms in the oxygen range from 0.09*%* to 2.85*%*, which corresponds to 1.2 to 37 *μ*M dissolved oxygen. The earlier estimate of 3 – 5 *μ*M [2] for preferred oxygen is very rough. It was determined first using microelectrodes that had a sensitivity limit of 1% oxygen [10]. A complimentary method used a gas proportioner to control the oxygen concentration in a gas mixture flowing into a gas chamber in which the capillary tubes were placed. The front of the band was adjacent to the meniscus when the oxygen concentration, determined by the gas proportioner, was 0.5% and the band dissipated and disappeared at oxygen concentrations lower than 0.05% in the gas mixture. While the method provided approximate numbers on oxygen concentrations, it is neither sensitive nor accurate. Furthermore, one would expect that manipulating the oxygen concentration in the gas mixture flowing into the cell would affect the aerotaxis response and bias the outcome. Thus, the oxygen range we obtained here via the model appears reasonable.

A simulation using parameter values from Mazzag et al. [15] produces a band that keeps moving over time and does not stabilize, shown in Fig. 2c d. The parameters that differ from those in Table 1, are: *B*_*o*_= 1×10^8^ cells/ml, *K*= 1×10^−9^*μ*M ml /s/cell, *v*=40 *μ*m/s, *F*_*max*_=0.5/s, *F*_*min*_=0.1/s, $\widehat {C}_{max}$=5%, *C*_*max*_=0.5%, and also the (Henry’s Law) factor for conversion of oxygen% in air to *μ*M dissolved oxygen in water: 1200 *μ*M whereas we use factor 1300. Crucial parameters for getting a stable band are *K*, *B*_*o*_, *C*-switches. Further replacing other parameters with ours eventually leads to Fig. 2a b that matches experimental measurements.

It should be noted that the model is capable of producing a great variety of band behaviors: wavy sides / smooth but moving / steady but not smooth / steady and smooth, at various locations, with various widths, all depending *on combinations of parameters*. No single parameter can account for any particular effect. Unwildly band behavior is not normally observed in experiments, so if they arise during simulations they are deemed unphysical, indicating poor parameters.

Parameter idendification is an “ill-posed” problem mathematically, typically uniqueness of solution and/or continuous dependence on data break down. Nevertheless, seeking *C*-switches to match *both location and width* of the experimentally measured band seems to constrain the system to have unique solution or no solution. In our extensive simulations we are able to find either *only one* combination or none at all.

### Band sensitivity on parameters

Having validated the model on experimental data, we present parametric studies on the main parameters *B*_*o*_, *C*_*o*_, *K*, *v*, *F*_*max*_, *F*_*min*_, and on the *C*-switches: $\widehat {C}_{max}$, *C*_*max*_, *C*_*min*_, $\widehat {C}_{min}$, to see how increasing or decreasing each one affects the band location and width, and by how much. The results are listed in Table 2.

For ease of comparison, the base value (from Table 1) of each parameter is listed, that produces location 406, width 132 *μ*m. Only one parameter is varied at a time, with all others at their base values. The % changes are relative to base values. It should be noted that the sensitivities shown in Table 2 are *local* about the base values. They may be different about some other base state.

In the following subsections we discuss some of the rows in Table 2 to point out the meaning of the entries. Similar considerations apply to the rest of the parameters in Table 2.

### Effect of bacterial density *B*_*o*_

Band location and width depend strongly on the total bacteria density. Inreasing *B*_*o*_ by 50% of base value to 1×10^9^ cells/ml, the band formed closer to the meniscus, at 352 *μ*m (−13% change), with narrower width (114 *μ*m, −14% change). The maximum of the bacterial distribution in the band (Bpeak, in column 7), is now 2.6, meaning 2.6×*B*_*o*_, a bit lower than the 2.9 peak of the base case. The entry “smooth” in column 8 refers to the shape of band sides in a plot like Fig. 2a. Changing *B*_*o*_ by −50*%* to *B*_*o*_=3×10^8^ cells/ml, the band formed much further (at 582 *μ*m, +43% change), with wider width (156 *μ*m, +18% change). Thus, band position and width are both decreasing functions of *B*_*o*_, with location being affected more strongly than width, especially at lower *B*_*o*_. These are in agreement with general experimental observations.

### Effect of Oxygen at the capillary opening, *C*_*o*_

Band location depends strongly on the oxygen concentration at the meniscus opening, which affects the oxygen gradient into the capillary. When *C*_*o*_ was increased to 30%, the band formed much further, at 527 *μ*m (+30% change), but with narrower width 122 *μ*m (−8% change). The entry “ ∼smooth” in column 8 means the band sides are mostly smooth but with a few step-like movements. When *C*_*o*_ was halved to 10%, the band formed much closer to the meniscus, at 227 *μ*m (−44% change), with unchanged width. The entry “smooth >220s” means the band sides show some step-like movements early on and become smooth after time 220s. Thus, band position is an increasing function of *C*_*o*_, but band width is little affected by *C*_*o*_. The effect of increasing/decreasing *C*_*o*_ on band location is as one would expect: increasing *C*_*o*_ raises the oxygen concentration profile *C*(*x*), so the switch values *C*_*max*_, *C*_*min*_ and the band occur further to the right.

### Effect of consumption rate *K*

Band location and width depend considerably on oxygen consumption rate, as is to be expected. When *K* was increased by 50% to *K*=6×10^−9^*μ*M ml/s cell, the band formed closer to the meniscus at 344 *μ*m (−15% change), with narrower width 114 *μ*m (−14% change). When *K* was decreased 50% to *K*=2×10^−9^*μ*M ml/s cell, the band formed much further away, at 547 *μ*m, a considerable change of +32%. It had wider width of 149 *μ*m (+13% change). Thus, band position and width are decreasing functions of oxygen consumption rate *K*, with location being affected much more than the width. Again, the predicted behavior aligns with experimental observations.

## Discussion

The ability to navigate gradients of oxygen is key to regulate metabolic activities of bacteria with a range of lifestyles. It is thus not surprising to observe that aerotaxis is a widespread behavior in bacteria and Archaea [2].

Several mathematical models have been developed to recapitulate the movement of bacteria in oxygen gradients. The models developed for bacteria that track higher concentrations of oxygen such as *B. subtilis* [16] or which prefer lower oxygen concentrations such as *Desulfovibrio desulfuricans* [17] are not appropriate for *A. brasilense* because the aerotaxis strategy of these organisms is distinct. *B. subtilis* detects oxygen directly and navigates toward elevated oxygen concentrations while *D. desulfuricans* is a strict anaerobe that forms a band at the oxic-anoxic interface with the band being far less stable than that observed for *A. brasilense*.

When we attempted to use the previously developed model for *A. brasilense* aerotaxis band formation by Mazzag et al. [15], we could not produce a stable aerotactic band, despite this feature being characteristic of the *A. brasilense* aerotaxis response [10, 11]. The model and experimental values used here provide a robust model that captures all significant features of *A. brasilense* aerotaxis band formation.

Our model predicts that cell density (*B*_*o*_), oxygen concentration at the meniscus (*C*_*o*_), and oxygen consumption rate (*K*) have significant effect on the *location* of the aerotactic band, but speed does not. On the other hand, *width* of the band is most sensitive to cell density (*B*_*o*_) and speed (*v*), but not to *C*_*o*_. In addition to experimental data validating at least some of these observations here, Barak et al. [18] demonstrated that increasing the oxygen concentration available at the capillary opening delayed band formation, and led to an increase in the number of attracted bacteria to the band, i.e., the band became thicker over time.

The tight aerotactic band formed by *A. brasilense* in gradients of oxygen depends on the abilty to sense oxygen as both an attractant and a repellent. *A. brasilense* senses very low or very high oxygen concentrations as repellents and motile cells navigate the gradients to stay away from these two strong repellents to locate themselves where oxygen is an attractant [10]. These opposing behaviors are captured in the model described here and by our experimental data indicating a very high probability of reversal in swimming direction for cells within the band.

Determining the *C*-switch values computationally, captures the narrow range of oxygen concentrations the bacteria prefer to congregate in, forming an aerotactic band. As this is difficult to do reliably in experiments, it is a major advantage of the model and approach described here. In the validated model the band forms between 1.2 and 37 *μ*M dissolved oxygen. Given our findings and observed effects of respiration rates and density, we expect this range to vary with experimental conditions.

One would expect fluid mixing induced by swimming cells to increase the diffusion coefficient of oxygen in water; the issue is how significant it would be. In a rather thorough paper on the subject, Kasyap et al. [19] estimated the hydrodynamic diffusivity induced by swimming bacteria, and conclude that “*bacteria induced mixing is irrelevant for small molecules*”. Indeed, their formula for hydrodynamic diffusivity (for oxygen, using our parameter values) yields 0.7 *μm*^2^/s inside the band and 0.9 *μm*^2^/s outside the band. These are indeed negligible compared to the molecular diffusivity *D*=2000 *μm*^2^/s of oxygen in water. Motivated by this question, in Table 2 we examine the effect of a large ±25*%* change in *D* to gauge uncertainty. It turns out that a large +25*%* increase of *D* would result in a rather modest +10% increase in band location, and would have no effect on width. (The case of −25*%* change is included for completeness, it is not expected to arise).

## Conclusions

A mathematical model for aerotaxis band formation was presented and validated on experimental data for *Azospirillum brasilense*. A spatial gradient assay for aerotaxis and cell tracking provide values for swimming speed and reversal frequencies, which are parameters in the model. Four other model parameters (that cannot be measured experimentally) were determined computationally so as to match measured band location and width. With these parameters, the model captures all significant features of *A. brasilense* aerotaxis band formation. The simulation reveals that wild-type Sp7 *A. brasilense* forms the band in the range of 1.2 to 37 *μ*M dissolved oxygen.

Parametric studies predict that band location depends strongly on cell density (*B*_*o*_), oxygen concentration at the meniscus (*C*_*o*_), and oxygen consumption rate (*K*), but not on swimming speed (*v*). On the other hand, width of the band is most sensitive to *B*_*o*_, *v*, and *K*, but not to *C*_*o*_.

## Methods

### Strain growth conditions

The motile soil alphaproteobacterium *A. brasilense* strain Sp7 ([20]) was used in these experiments. *A. brasilense* cells were routinely grown in liquid MMAB (Minimal Medium for *A*zospirillum *b*rasilense [21]), supplemented with malate (10 mM) and ammonium chloride (18.7 mM), as previously described [14]. For Colony Forming Units (CFU) counts, liquid cultures were grown to the desired optical density at 600 nm (OD_600_). One ml aliquots were taken and serially diluted 10-fold and plated on TY medium (Tryptone 10 g/l, Yeast Extract 5 g/l) with ampicillin (200 *μ*g/ml).

### Spatial gradient assay for aerotaxis

Cells were grown to an OD_600_ of 0.4 - 0.6 (exponential phase of growth) in MMAB supplemented with malate and ammonium. Cultures were washed 3 times with chemotaxis buffer and standardized to an OD_600_ of 0.5 [22]. One ml of this culture of motile cells were gently washed with sterile chemotaxis buffer by centrifugation (5000 rpm for 3 minutes) and resuspended in 100 *μ*l chemotaxis buffer containing malate. Over 95% of cells remained motile under these conditions. Cells were transferred to an optically flat microcapillary tube (inner dimensions 0.1 ×2×50 mm, Vitro Dynamics, Inc., Rockaway, NJ) by immersing a capillary tube into the suspension of motile cells. The cells were equilibrated in a gas perfusion chamber with N_2_ gas for 3 minutes, then air (21% oxygen) was introduced, and aerotactic band formation was visualized under a light microscope at 4 × magnification, and videotaped at 30 fps. Upon formation of a stable band [13], band location was measured at mid-height of the capillary from (surface of) the meniscus to center of the band; band width was also measured at the same mid-height. Time to stable band formation was also measured.

Snapshots of band formation in such a spatial gradient assay are shown in Fig. 1. The band forms very fast and stabilizes within a couple of minutes.

### Single cell tracking

To determine the swimming reversal frequency and swimming speed of cells within and outside the aerotactic band, a digital recording (at 40 × magnification) of the aerotactic band formed by wild type strains under the conditions described above was analyzed using CellTrak (Santa Rosa, CA), following the procedure described in [11]. A minimum of 100 individual tracks were analyzed and the average values as well as minimum and maximal values were determined from these data sets and used for mathematical modeling.

## Mathematical Model

Various modeling approaches for chemotaxis exist: Ordinary Differential Equation (ODE) models for signaling pathways [23–25]; Partial Differential Equation (PDE) models of various types for chemotactic movement, most commonly Keller-Segel type models [26]; stochastic models of various types [27–29]; and agent-based models [30, 31].

The most extensively studied *mathematical* models for chemotaxis are *Keller-Segel* type models, named after the 1971 work of Evelyn Keller and Lee Segel [26], even though similar models were derived already by C.S. Patlak in 1953 [32]. Such models describe evolution of bacterial density by a parabolic PDE involving an anti-diffusion “chemotaxis term” proportional to the gradient of the chemoattractant, thus allowing movement up-the-gradient, the most prominent feature of chemotaxis. It has been shown that in 2 and higher (space) dimensions, under certain conditions, finite-time blow-up may occur which is clearly unphysical (sometimes interpreted as “overcrowding”) [33]. An excellent summary of mathematical results on Keller-Segel models up to 2004 can be found in [34, 35].

The type of model employed here was initially formulated for chemotaxis by Lee Segel [36, 37], and it is more physical (and more "primitive", in the sense that under appropriate assumptions it reduces to the Keller-Segel model). It was adapted for aerotaxis by Mazzag et al. [15] to model *energy taxis* [10, 11, 38]. A great advantage of the model is that it incorporates experimentally measurable parameters, namely swimming speed and reversal frequencies, as it will be described below. While [15] captured some of the features of the aerotaxis response of *A. brasilense*, it failed to produce a stable (not moving) aerotactic band, which is typical of that formed by *A. brasilense*.

Below, we present in full detail the basic mathematical model, which is a somewhat simplified version of [15], and then we mention some features of our numerical implementation.

### Swimming of the Bacteria

We formulate a system of partial differential equations that describe movement of bacteria whose reversal frequency is regulated by local oxygen concentration. We consider one-dimensional movement (along the x-axis) in an interval 0≤*x*≤*S*. The advection terms describe the directed swimming of bacteria with speed *v*, while the reaction terms denote the turning of bacteria at frequencies *f*_*RL*_ and *f*_*LR*_. *R*(*x*,*t*) and *L*(*x*,*t*) are the number (densities) of right-moving and left-moving bacteria at position *x* and time *t*, respectively.

1 $$  \frac{\partial R(x,t)}{\partial t} + v \frac{\partial R(x,t)}{\partial x} = -f_{RL} \: R(x,t)+f_{LR} \: L(x,t),  $$

2 $$ \frac{\partial L(x,t)}{\partial t} - v \frac{\partial L(x,t)}{\partial x} = +f_{RL} \: R(x,t)-f_{LR} \: L(x,t),  $$

where *v* is the (constant) swimming speed, *f*_*RL*_ and *f*_*LR*_ are the probabilities with which bacteria reverse their direction from to Right to Left and from Left to Right, respectively, given by

3 $$ f_{RL} = \left\{ \begin{array}{rl} F_{max} & \text{if} \quad \widehat {C}_{min} < C < {C}_{max} \,, \\ F_{min} & \text{if} \quad C<\widehat C_{min} \quad \text{or} \quad C>{C}_{max} \,, \end{array} \right.  $$

4 $$ f_{LR} = \left\{ \begin{array}{rl} F_{max} & \text{if} \quad {C}_{min} < C <\widehat C_{max} \,, \\ F_{min} & \text{if} \quad C<{C}_{min} \quad \text{or} \quad C>\widehat C_{max} \,. \end{array} \right.  $$

**Fig. 3:**
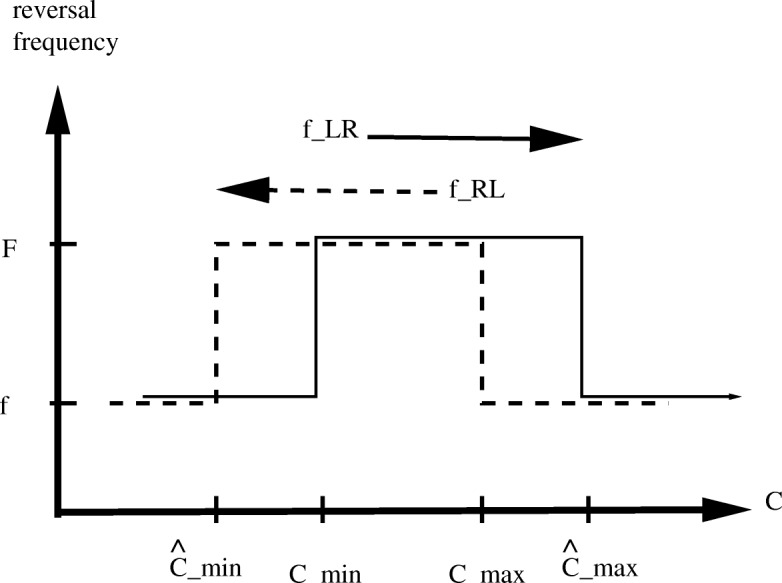
Reversal frequency of right swimming (solid line) and left swimming (dashed line) cells, depicting formulas (3) and (4), for setting *f*_*RL*_ and *f*_*LR*_ in the model

Here *F*_*max*_ and *F*_*min*_ are maximum and minimum reversal frequencies, respectively, and $\widehat {C}_{min} < {C}_{min} < {C}_{max} < \widehat {C}_{max}$ are specified switch values of oxygen concentration *C* at which frequencies change from low *F*_*min*_ to high *F*_*max*_ and vice versa. The formulae are depicted in Fig. 3.

In our implementation, we actually use different values for *F*_*max*_ inside and outside the band, which are found experimentally, see Table 1. The concentration of bacteria, *B*(*x*,*t*), is the total number of right- and left-moving cells:

5 $$ B(x,t)=R(x,t)+L(x,t).  $$

Cell reproduction is much slower than band formation time-scale and it is ignored. Initially, *R*(*x*,0)=*R*_*o*_(*x*) and *L*(*x*,0)=*L*_*o*_(*x*) in [0,*S*], for some initial distributions *R*_*o*_(*x*) and *L*_*o*_(*x*). At the left boundary all left-moving cells turn to the right, and at the right boundary all right-moving cells turn to the left: *R*(0,*t*)=*L*(0,*t*) and *R*(*S*,*t*)=*L*(*S*,*t*). These boundary conditions ensure that there is no depletion of bacteria, thus the total number of bacteria in the capillary [0,*S*] remains constant and equal to the initial number

6 $$ \int_{0}^{S} B(x,t) dx = const. = B_{o} = R_{o} + L_{o}.  $$

### Diffusion of Oxygen

The oxygen concentration *C*(*x*,*t*) in the capillary [0, *S*] is determined by a diffusion-reaction equation that accounts for the consumption of oxygen by the bacteria:

7 $$ \frac{\partial C(x,t)}{\partial t} = D \frac{\partial^{2} C(x,t)}{\partial x^{2}} - K \, \theta(C(x,t)) \, B(x,t) \,,  $$

where *B*(*x*,*t*) is the concentration of bacteria (Eq. 5), *K* is the rate of consumption of oxygen by bacteria, and *D* is diffusion coefficient of oxygen in water. To ensure there is no consumption after oxygen depletion, *θ*(*C*) is set as

8 $$ \theta(C(x,t)) = \left\{ \begin{array}{rl} 1 & \text{if} \;\; C(x,t) > 0, \\ 0 & \text{if} \;\; C(x,t) \leq 0. \end{array} \right.  $$

Initially there is no oxygen in the capillary, so the initial condition is

9 $$ C(x,0) = 0 \quad \text{for all} \quad 0 \leq x \leq S.  $$

At the open end *x*=0 the oxygen concentration is a specified value *C*_*o*_, while the other end of the capillary is sealed (with wax) to prevent oxygen from entering or leaving. Thus the boundary conditions at *x*=0 and *x*=*S* are

10 $$ C(0,t) = C_{o}\,, \quad \quad \frac{\partial C(x,t)}{\partial x} = 0 \;\; \text{ at} \; x=S \,.  $$

### Numerical Implementation

The system of partial differential eqs. (1), (2), (7) was undimensionalized and solved numerically in Fortran 90. The advection equations were discretized with an upwind Finite Volume scheme and forward Euler time discretization. The diffusion equation was also discretized by Finite Volumes with forward Euler time discretization.

In the simulations, we used capillary length *S*=5 mm, which is already far away from where the band forms and does not affect the numerical results. The capillary, occupying the interval [0, *S*] was discretized into 640 control volumes (128 per mm), and the time-step was chosen judiciously and adaptively to ensure numerical stability and non-negative concentrations.

We note here some features in our implementation and differences with Mazzag et al. [15].

(1) In conformance with measurements, reversal frequency is higher inside the band instead of lower. Thus Fig. 3 is a “flipped” version of Fig. 2c in [15]. (2) Reversal frequencies are applied separately inside the band and outside the band (different values for *F*_*max*_ may be used inside and outside the band). This was motivated by experimental measurements, and necessitated calculation of band location at each time-step in the numerical implementation. The band is calculated from the bacterial distribution *B*(*x*,*t*_*n*_) at each time-step *t*_*n*_, as FWHM (Full Width at Half Maximum), a common practice in many fields. This is done by intersecting the density profile by a horizontal line at half-maximum to determine the left side (*xL*) and right side (*xR*) of the band at time *t*_*n*_, which are plotted in Fig. 2a. (3) In the simulations we use much finer space grid (128/mm instead of 40/mm used in [15]) which noticeably affects the calculated band location.

## Abbreviations

CCW: Counter clock wise rotation of flagellar motor
CFU: Colony forming units
CW: Clock wise rotation of flagellar motor
MMAB: Minimal medium for azospirillum brasilense
ODE: Ordinary differential equation
OD_600_: Optical density at 600 nm
PDE: Partial differential equation
